# Small Kinetochore Associated Protein (SKAP) Promotes UV-Induced Cell Apoptosis through Negatively Regulating Pre-mRNA Processing Factor 19 (Prp19)

**DOI:** 10.1371/journal.pone.0092712

**Published:** 2014-04-09

**Authors:** Shan Lu, Renxian Wang, Congli Cai, Junbo Liang, Longchang Xu, Shiying Miao, Linfang Wang, Wei Song

**Affiliations:** State Key Laboratory of Medical Molecular Biology, Dept. of Biochemistry and Molecular Biology, Institute of Basic Medical Sciences Chinese Academy of Medical Sciences, Peking Union Medical College, Beijing, China; University of Cincinnati, College of Medicine, United States of America

## Abstract

Apoptosis is a regulated cellular suicide program that is critical for the development and maintenance of healthy tissues. Previous studies have shown that small kinetochore associated protein (SKAP) cooperates with kinetochore and mitotic spindle proteins to regulate mitosis. However, the role of SKAP in apoptosis has not been investigated. We have identified a new interaction involving SKAP, and we propose a mechanism through which SKAP regulates cell apoptosis. Our experiments demonstrate that both overexpression and knockdown of SKAP sensitize cells to UV-induced apoptosis. Further study has revealed that SKAP interacts with Pre-mRNA processing Factor 19 (Prp19). We find that UV-induced apoptosis can be inhibited by ectopic expression of Prp19, whereas silencing Prp19 has the opposite effect. Additionally, SKAP negatively regulates the protein levels of Prp19, whereas Prp19 does not alter SKAP expression. Finally, rescue experiments demonstrate that the pro-apoptotic role of SKAP is executed through Prp19. Taken together, these findings suggest that SKAP promotes UV-induced cell apoptosis by negatively regulating the anti-apoptotic protein Prp19.

## Introduction

Apoptosis plays an important role in regulating homeostasis, and failures in the regulation of apoptosis can lead to many human diseases, such as cancer, autoimmune disorders, and neurodegenerative disorders [1]. Studies performed over the past few years have demonstrated that there are two major apoptotic pathways, the extrinsic and intrinsic pathways [2]. The extrinsic pathway is death receptor-mediated apoptosis, initiated by members of the TNF superfamily, including TNFα and TRAIL [3], [4]. The intrinsic pathway triggers apoptosis in response to DNA damage, cell cycle checkpoint defects or other types of severe cell stresses [5]. Apoptosis inducing agents such as ultraviolet light (UV) and Staurosporine (STS) mainly induce apoptosis through the intrinsic pathway [6], [7]. The extrinsic and intrinsic pathways both end at the execution phase. Caspase-3, caspase-6, and caspase-7 function as “executioner” caspases, cleaving substrates including PARP, cytokeratins and others, and ultimately causing the morphological and biochemical changes seen in apoptotic cells [8]–[10].

Small kinetochore associated protein (SKAP) was originally named HSD11 when it was cloned from a human testis cDNA library and deposited in GenBank (GenBank access number AY652615). Subsequent studies identified HSD11 as a G2-induced protein that could cooperate with kinetochore and mitotic spindle proteins to regulate mitosis [11], and it was then renamed SKAP, for Small Kinetochore Associated Protein [12]. The interaction between SKAP and astrin in the kinetochore has been reported to be essential for accurate mitosis [13], [14]. Meanwhile, SKAP interacts with CENP-E to orchestrate accurate chromosome movement in mitosis [15]. A recent study also found that SKAP constitutes a dynamic link between the spindle microtubule plus-ends and mitotic chromosomes to achieve faithful cell division [16]. Despite the important role SKAP plays in mitosis, other biological functions of SKAP, such as in apoptosis, have not been studied.

In this study, we show for the first time that SKAP promotes UV-induced cell apoptosis. We performed a tandem affinity purification/mass spectrometry (TAP/MS) experiment and identified the multi-functional protein Pre-mRNA processing Factor 19 (Prp19) as a SKAP interactor. Further study revealed that SKAP could negatively regulate Prp19 protein levels and that the apoptosis promoting effect of SKAP could be rescued by Prp19. Collectively, our results suggest that SKAP promotes UV-induced cell apoptosis by antagonizing Prp19.

## Materials and Methods

### Cell Culture and Treatment

HeLa, HEK-293T and HCT116 cells were obtained from the Cell Resource Center of Peking Union Medical College (PUMC). HeLa and HEK-293T cells were cultured in Dulbecco’s Modified Eagle’s Medium (DMEM) supplemented with 10% fetal bovine serum (FBS); HCT116 cells were cultured in Iscove’s Modified Dulbecco’s Medium (IMDM) supplemented with 10% FBS. All of the cell lines were cultured in a 5% CO_2_ incubator at 37°C, and they were passaged every 2–3 days with 0.5 mg/ml trypsin (1∶250) and 0.53 mM ethylenediaminetetraacetic acid (EDTA). To induce apoptosis, HeLa cells were treated with 20 ng/ml TNFα, 50 ng/ml TRAIL or 0.2 μM Stauriprione for 12 hours. For ultraviolet light (UV) treatment, DMEM was displaced with PBS, and cells were exposed to 40 J/m^2^ UV irradiation in a GS Genelinker UV chamber (Bio-Rad). The cells were then maintained in DMEM and harvested at indicated times.

### Immunofluorescence Microscopy

SKAP immunostaining was performed as described previously [17]. Briefly, HeLa cells were plated on coverslips in DMEM medium and cultured for 24 hours. The cells were rinsed with PBS, fixed in 4% formaldehyde solution for 10 min, and then extracted in 0.5% Triton X-100 for 10 min. After being incubated in PBS containing 3% BSA at 37°C for 30 min, the cells were incubated with polyclonal antibodies against SKAP (1∶200) or pre-immune serum at 37°C for 30 min and incubated in FITC-conjugated goat anti-rabbit IgG (1∶200) at 37°C for 30 min. Finally, the cells were treated with 2 μg/ml propidium iodide (PI) and 20 μg/ml RNaseA at 37°C for 30 min. The cells were examined using a confocal laser scanning microscope (Leica TCS NT).

### Plasmids, siRNA and Transfection

Plasmids were constructed using standard cloning techniques. The coding regions of SKAP and Prp19 were amplified by PCR from a Human cDNA library and cloned into either a pcDNA3.1-Flag vector or a pcDNA3.1-Myc vector. For SKAP, the forward primer was 5′-ACATCTTCATACGGGAGGCTT-3′ and the reverse primer was 5′-TCACTGGCTTCGTCCTTACC-3′; for Prp19, the forward primer was 5′-TAAGAATTCATGTCCTAATCTGCTCCATCTCTAAC-3′ and the reverse primer was 5′-TAAGCGGCCGCCTACAGGCTGTAGAACTTGAGGCTTC-3′. All of the siRNAs were synthesized by Shanghai GenePharma Company. The sequences used to target SKAP were #1∶5′GAGUCCGAUUCCUAGAACATT 3′; #2∶5′-GAAAGAGUCCGAUUCCUAGUUT-3′. The sequences used to target Prp19 were #1∶5′-GCCACUAUCAGGAUUUGGUTT-3′; #2∶5′- CUUGAAGGAACGUACUAAUTT-3′; #3∶5′-GCCAAGUUCAUCGCUUCAATT-3′. The sequence used for the siRNA negative control was 5′-UUCUCCGAACGUGUCACGU-3′. Plasmid transfection was performed with Lipofectamine 2000 (Invitrogen), and siRNA transfection was performed with Lipofectamine RNAiMAX (Invitrogen) following the protocol suggested by the manufacturer.

### Quantitative Real-time-PCR (qRT-PCR)

Total RNA from HeLa, HEK-293T and HCT116 cells was extracted with TRIzol reagent (Invitrogen), and 1 μg of isolated total RNA was converted to cDNA using the Roche Transcriptor First-Strand cDNA Synthesis kit (Roche). Power SYBR green master mix (Applied Biosystem) was added to cDNA samples that were then subjected to qRT-PCR using the Step-One Realtime PCR system. Relative Prp19 mRNA levels were normalized against the housekeeping gene GAPDH. The primers for qRT-PCR were as follows. A forward primer, 5′-ATGTCCCTAATCTGCTCCATCT-3′, and reverse primer, 5′-GAGCCGCCGCTCATAAACA-3′, was used to amplify Prp19. A forward primer 5′-TGAGTACGTCGTGGAGTCCA-3′, and reverse primer, 5′-TAGACTCCACGACATACTCA-3′, was used to amplify GAPDH.

### Production of Retrovirus and Generation of HEK-293T Stable Cells

The production of retrovirus has been described previously [18]. Briefly, retroviral vectors expressing either Flag-StrepII-SKAP or Flag-StrepII-RFP were co-transfected with helper plasmids containing gag-pol and env into HEK-293T cells. After 48 hours, the supernatant was collected, filtered through a 0.45 μm filter and concentrated by centrifuging the solution at 18,000 rpm for 3 hours at 4°C. To generate HEK-293T cells stably expressing either Flag-StrepII-SKAP or Flag-StrepII-RFP recombinant proteins, cells cultured in a 35 mm dish were infected with the concentrated retrovirus followed by one week of selection with 2 μg/ml puromycin (Sigma).

### Immunoblot Analysis

At the indicated times after transfection, cells were washed with PBS, harvested, lysed in a buffer containing 50 mM Tris-HCl [pH 6.8], 10% glycerol and 2% SDS, and quantified using the BCA protein assay reagent (Pierce). The cell extracts were separated on a 10% or 12% SDS-PAGE gel and then electrophoretically transferred to a PVDF membrane (GE Healthcare) according to standard protocols. The membrane was blocked in 5% skim milk for 1 hour at room temperature and then incubated overnight with the indicated antibodies at 4°C. The membrane was incubated with an anti-mouse or an anti-rabbit HRP-IgG (Santa Cruz) for 1 hour at room temperature. Chemiluminescence was detected using an ECL blot detection system (Engreen). The antibodies used in this study were as follows: rabbit anti-SKAP and rabbit anti-Prp19 antibodies from Abcam; mouse anti-GAPDH, anti-histone H1 and mouse anti-β-actin antibodies from Santa Cruz; mouse anti-Caspase-7 antibody from MBL; mouse anti-Flag and mouse anti-Myc antibodies from Sigma; rabbit anti-PARP antibody from Cell Signaling Technology.

### Tandem Affinity Purification and Mass Spectrometry

Approximately 5×10^8^ HEK-293T cells (corresponding to five 150 mm culture dishes) stably expressing either Flag-StrepII-SKAP or Flag-StrepII-RFP were harvested and sonicated in NETN lysis buffer (20 mM Tris-HCl, pH 8.0; 100 mM NaCl; 0.5% Nonidet-P40; 1 mM EDTA) containing protease inhibitor cocktails (Roche). The cell lysates were centrifuged at 14,000 rpm for 1 hour. The supernatant was incubated with Strep Tag II agarose at 4°C for 1 hour, followed by three washes with NETN buffer to remove the contaminants. The bait proteins were eluted with desthiobiotin (IBA) buffer according to the supplier’s instructions. The elutions were then incubated with Flag-agarose at 4°C for 2 hours. The beads were pelleted and washed three times with NETN buffer, followed by incubation with 3×Flag peptides (Sigma) dissolved in NETN buffer to elute the bound proteins. The eluted proteins were resolved by 4–12% Bis-Tris NuPAGE gels (Invitrogen Life technologies) and visualized by Coomassie blue staining. The visible bands were excised from the gel, and the proteins were identified by mass spectrometry.

### Immunoprecipitation Assay

Cells were transiently transfected with pcDNA3.1-Flag-SKAP, or pcDNA3.1-Myc-Prp19 as indicated. After 48 hours, the cells were harvested and lysed in NETN buffer as previous described. Protein complexes were isolated using anti-Flag beads (Sigma, M2) or anti-Myc beads (Santa Cruz). The protein complexes were solubilized in 1.2×SDS loading buffer and analyzed by immunoblot.

### Flow Cytometry Analysis

HeLa cells were treated with the specified stimuli to induce apoptosis. At the indicated times, cells were incubated with 0.25% trypsin-EDTA at 37°C for 3 min, collected, washed twice with PBS and stained with 50 μg/ml propidium iodide (PI) and 50 μg/ml RNaseA in PBS at 37°C for 20 min. The DNA content of 10,000 cells was analyzed using a COULTER flow cytometer (EPICS-XL) with EXPO32-ADC software. The percentage of apoptotic cells (% of total cells) was analyzed using the program EXPO32-ADC, and the results are shown as a bar graph. Double staining with FITC-AnnexinV and PI was performed using the FITC-AnnexinV/PI Apoptosis Kit (MBL) according to the manufacturer’s recommendations, and the cells were analyzed using the Accuri C6 flow cytometer system.

## Results

### SKAP Encodes an Evolutionarily Conserved Nuclear Protein

**Figure 1:**
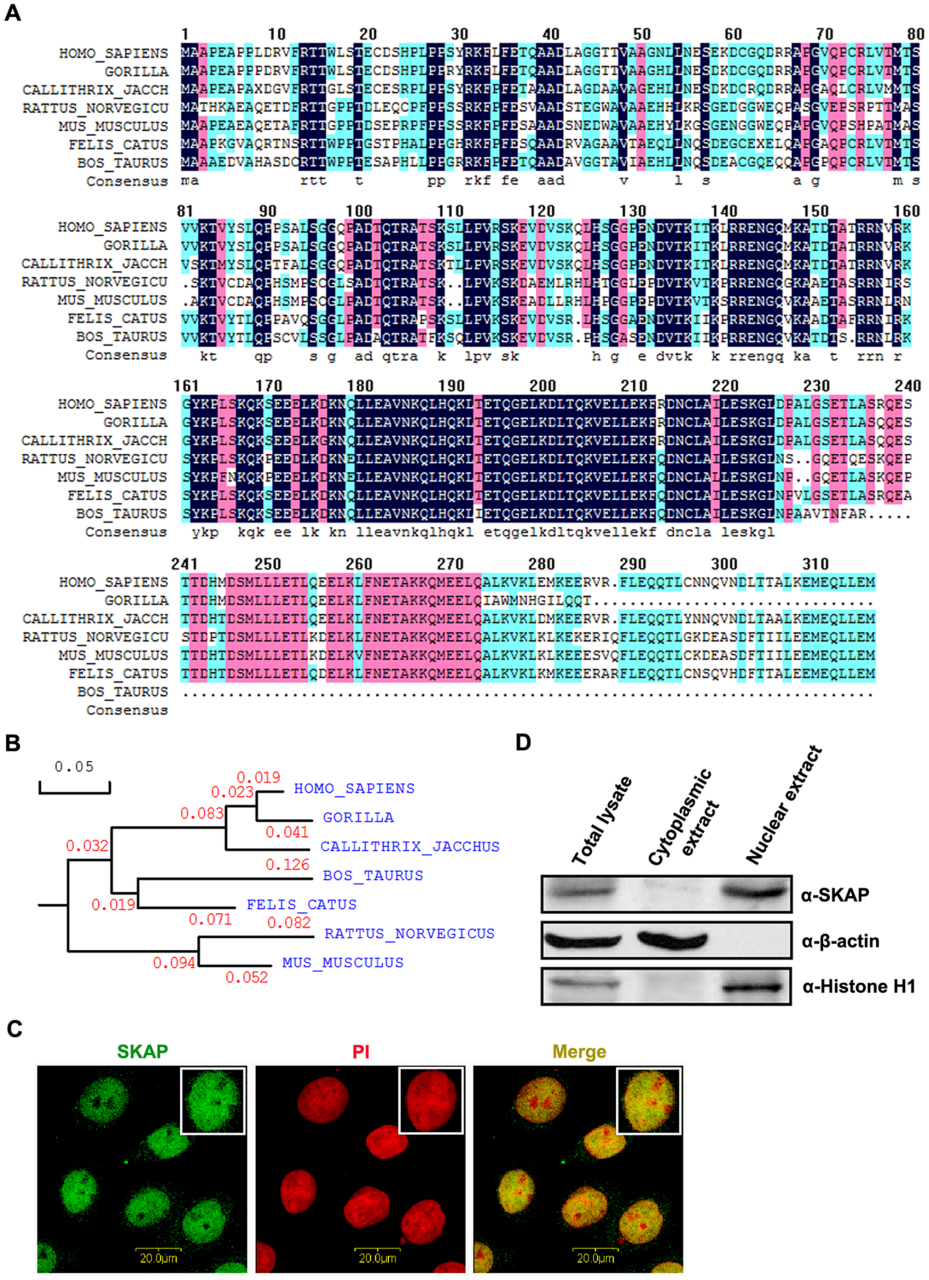
The evolutionary conservation and subcellular localization of SKAP. (A) Amino-acid sequence alignment of SKAP from different species. The alignment was performed by DNAMAN. Homology levels are highlighted in different colors. Black: 100%; Pink: 75%; Blue: 50%. (B) Phylogenetic analysis of evolutionary relationships among homologs of SKAP proteins from different species. (C) Immunofluorescence staining for SKAP subcellular localization. Endogenous SKAP localization was analyzed by immunofluorescence with an anti-SKAP antibody (Green). Nuclei were stained with PI (Red). Cells were visualized by confocal microscopy (Leica). Scale bars are 15 μm. (D) Immunoblot analysis of isolated cytoplasmic and nuclear proteins to determine SKAP subcellular localization. β-actin and histone H1 were used as a cytosolic marker and a nuclear marker, respectively.

We first performed an amino-acid sequence alignment of SKAP in seven species: *Homo sapiens*, *Gorilla*, *Callithrix jacchus*, *Rattus norvegicus*, *Mus musculus*, *Felis catus* and *Bos Taurus*. The amino-acid sequence of human SKAP is highly conserved with homologs in other organisms, with 97.82% identity to Gorilla, 90.51% to *Callithrix jacchus*, 67.56% to *Rattus norvegicus*, 68.49% to *Mus musculus*, 79.05% to *Felis catus, and* 75.56% to *Bos Taurus* (Figure 1A). The phylogenetic analysis also indicated that the SKAP gene has been evolutionarily well-conserved (Figure 1B). Next, we used immunofluorescence to observe the intracellular localization of endogenous SKAP in HeLa cells. As shown in Figure 1C, SKAP was exclusively localized in the nucleus. To further verify the nuclear localization of SKAP, cytoplasmic and nuclear proteins were isolated and immunoblotted. SKAP was exclusively expressed in the nucleus (Figure 1D). Collectively, these results indicate that SKAP is a phylogenetically conserved nuclear protein.

### Ectopic Expression of SKAP Promotes UV-induced Cell Apoptosis

**Figure 2:**
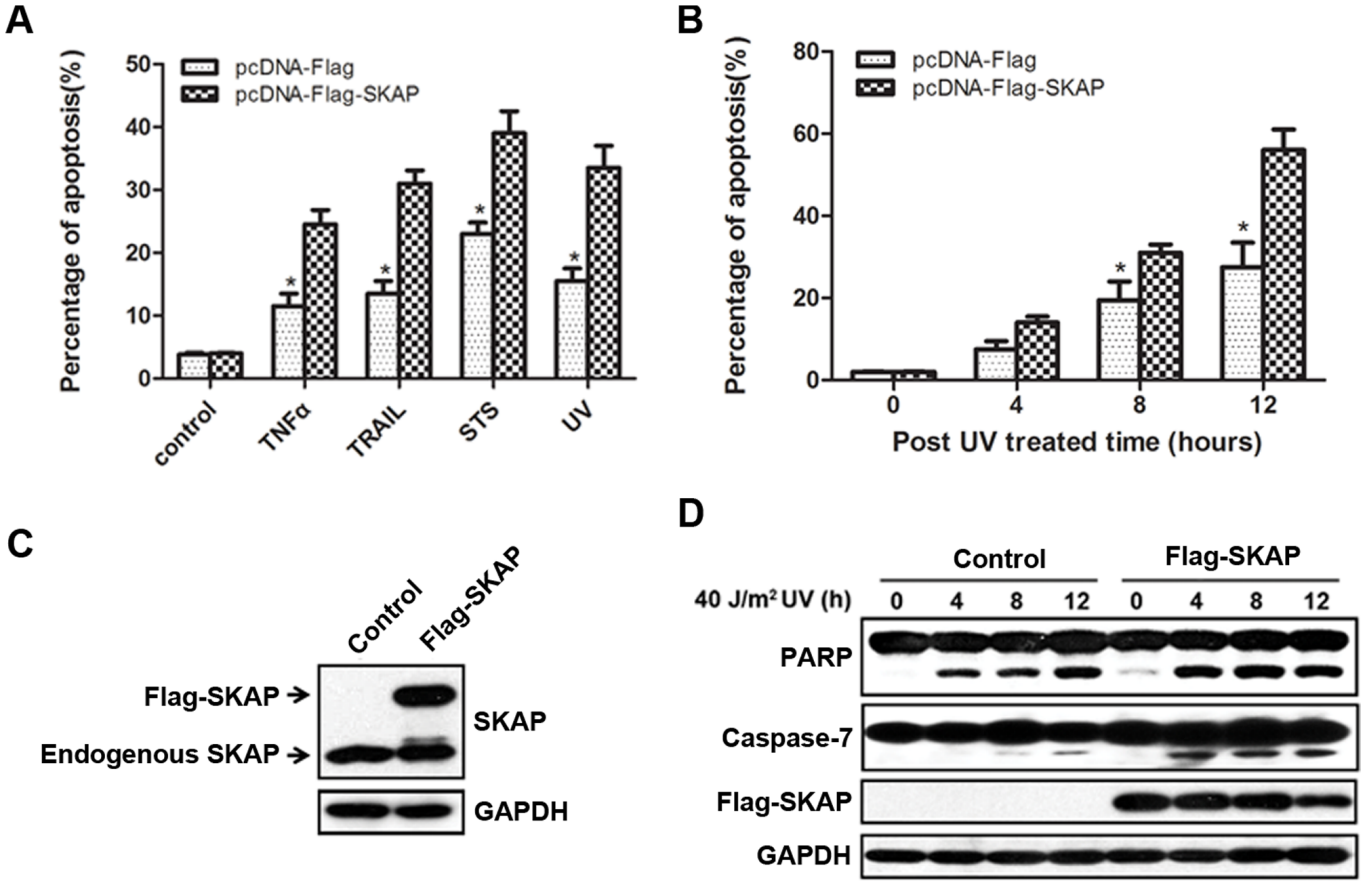
Ectopic expression of SKAP in HeLa cells promotes UV-induced apoptosis. (A) FACS analysis (PI incorporation) to analyze apoptosis of HeLa cells transfected with pcDNA-Flag or pcDNA-Flag-SKAP after treatment with TNFα (20 ng/ml), TRAIL (50 ng/ml), Staurosporine (STS, 0.2 μM) or 40 J/m^2^ UV exposure. The percentage of apoptotic cells (% of total cells) was determined using EXPO32-ADC, and the results are shown as a bar graph (*p<0.05). The values represent the mean ± SD from three independent experiments. (B) HeLa cells were transfected with pcDNA-Flag or pcDNA-Flag-SKAP and exposed to 40 J/m^2^ UV irradiation. FACS analysis was used to analyze apoptosis as described in (A). (C) Confirmation of SKAP expression in HeLa/Flag-SKAP stable cell line by immunoblot analysis. GAPDH was used as a loading control. (D) HeLa/Flag and HeLa/Flag-SKAP stable cells were treated with 40 J/m^2^ UV irradiation. The cell lysates were analyzed by immunoblot with the antibodies indicated. GAPDH was used as a loading control.

To study the effect of SKAP on cell apoptosis, HeLa cells were transiently transfected with Flag-SKAP or an empty vector, treated with TNFα, TRAIL, Staurosporine(STS) or UV irradiation, and then analyzed by FACS. As shown in Figure 2A, SKAP overexpression alone did not appear to induce apoptosis; however, the percentage of apoptotic cells increased significantly more than controls when cells transfected with Flag-SKAP were treated with four apoptotic stimuli. UV irradiation was selected as an apoptosis stimulus in the subsequent experiments. Our results indicate that SKAP overexpression promotes HeLa cell apoptosis in a time-dependent manner (Figure 2B). To further investigate the pro-apoptotic role of SKAP, we generated a HeLa cell line that stably expressed the Flag-SKAP recombinant protein at a level 1.5-fold higher than the endogenous SKAP level (Figure 2C). Within 8 h after exposure to UV irradiation, a greater number of cells became round and detached from the dish in HeLa cells stably expressing Flag-SKAP compared to control cells (data not shown). We next performed immunoblot analysis to validate the pro-apoptotic effect of SKAP. Following UV treatment, SKAP overexpressing cells showed a dramatic increase in the level of cleaved caspase-7 and cleaved PARP (Figure 2D). Taken together, these data demonstrate that overexpression of SKAP increases the sensitivity of HeLa cells to UV-induced apoptosis.

### SKAP Silencing Causes Resistance to UV-induced Cell Apoptosis

**Figure 3:**
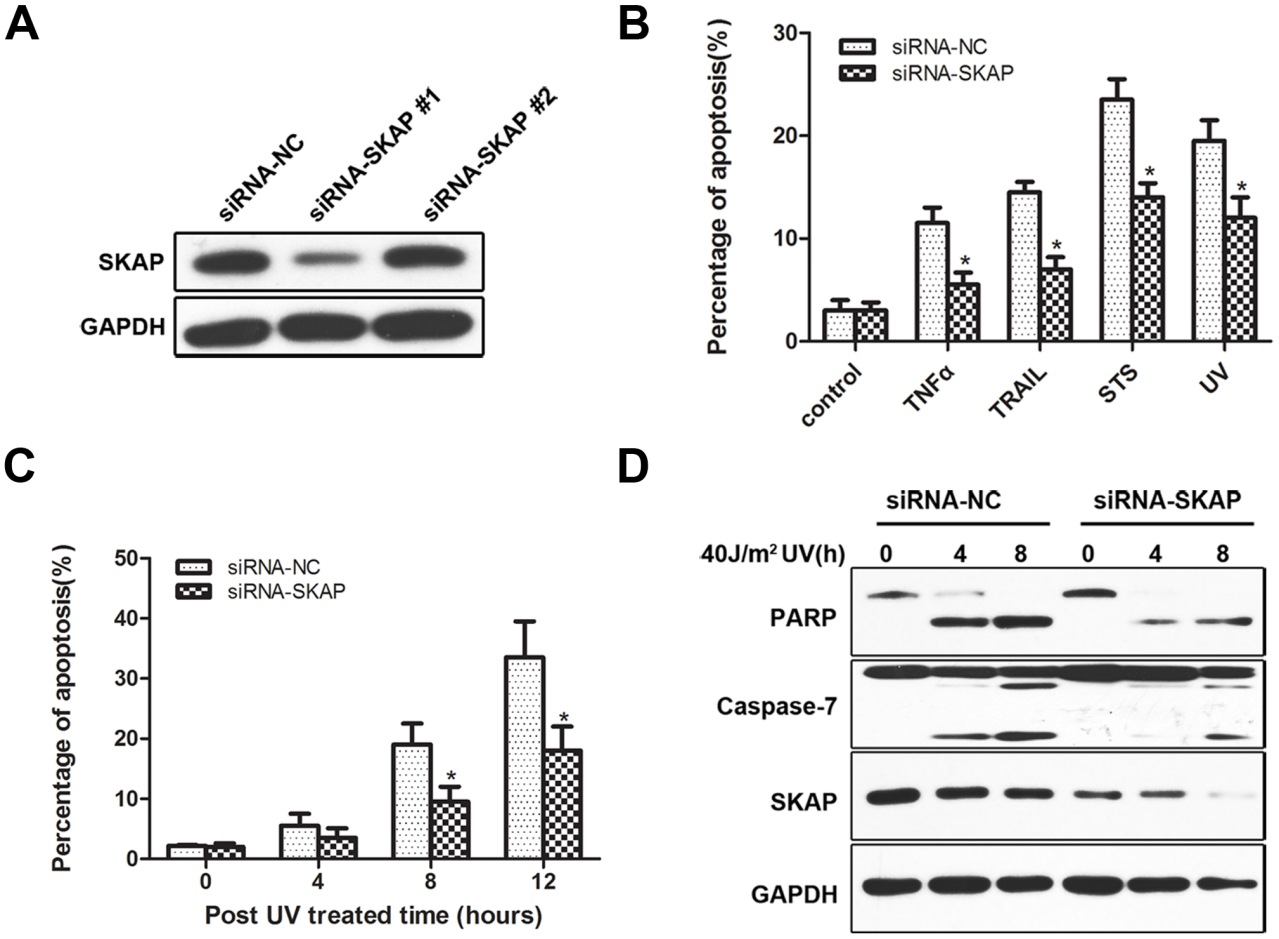
Knockdown of SKAP in HeLa cells inhibits UV-induced apoptosis. (A) The knockdown efficiency of siRNAs targeting SKAP was verified by immunoblot analysis. (B) HeLa cells were transfected with siRNA-NC or siRNA-SKAP #1 and treated with TNFα (20 ng/ml), TRAIL (50 ng/ml), Staurosporine (STS, 0.2 μM) or 40 J/m^2^ UV exposure. FACS analysis (PI incorporation) was used to analyze apoptosis as described in Figure 2(A). (C) HeLa cells were transfected with siRNA-NC or siRNA-SKAP #1 and exposed to 40 J/m^2^ UV irradiation. FACS analysis was used to assess apoptosis as described in Figure 2A. (D) HeLa cells were transfected with siRNA-NC or siRNA-SKAP #1 and treated with 40 J/m^2^ UV irradiation. The cell lysates were analyzed by immunoblot with the antibodies indicated. GAPDH was used as a loading control.

To further clarify the role of SKAP in UV-induced apoptosis, we down-regulated endogenous SKAP using RNA interference and observed its effect on cell apoptosis. Two small interfering RNA (siRNA) molecules targeting SKAP were introduced into cells to attenuate the expression of endogenous SKAP. Immunoblot analysis showed that siRNA-SKAP #1 effectively inhibited SKAP expression, and that construct was used in the following experiments (Figure 3A). HeLa cells transfected with siRNA-SKAP or siRNA-NC were treated with TNFα, TRAIL, Staurosporine or UV irradiation and then analyzed by FACS. As shown in Figure 3B, SKAP knockdown significantly suppressed the amount of cell apoptosis induced by all four stimuli. We next demonstrated that SKAP knockdown inhibited UV-induced cell apoptosis in a time-dependent manner (Figure 3C). Furthermore, we observed that knockdown of SKAP decreased the extent of UV-induced apoptotic morphology (data not shown). The immunoblot analysis revealed a marked decrease in the level of cleaved caspase-7 and cleaved PARP after UV stimulation (Figure 3D). Collectively, these results demonstrate that silencing of endogenous SKAP significantly inhibits UV-induced cell apoptosis.

### Identification of SKAP Interacting Proteins by Tandem Affinity Purification and Mass Spectrometry

**Figure 4:**
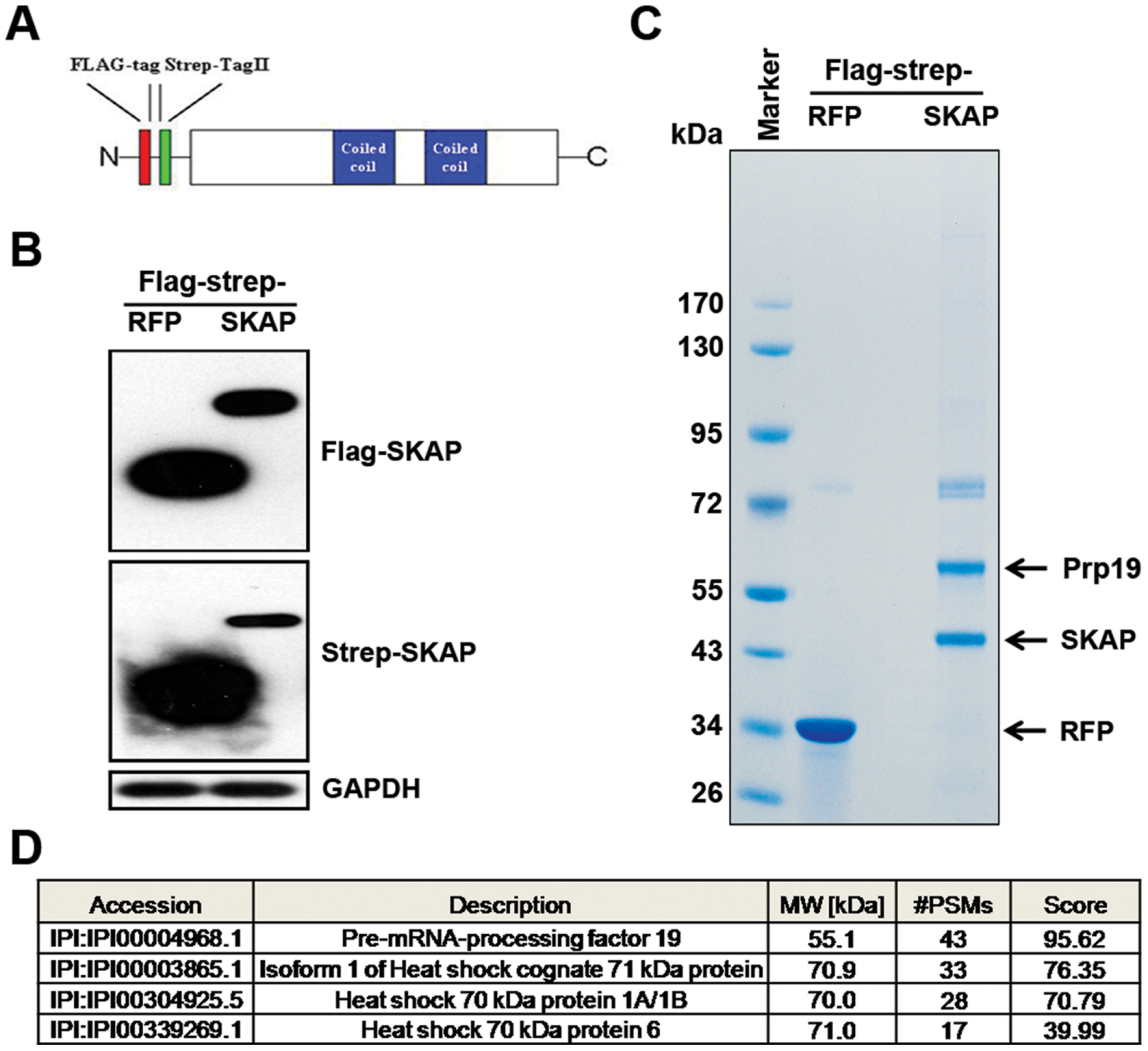
Identification of SKAP interacting proteins by TAP/MS. (A) Schematic of SKAP domain structure and the position of the Flag-strepII tag. (B) Confirmation of SKAP expression in HEK-293T/Flag-strepII-SKAP stable cell line by immunoblot analysis. GAPDH was used as a loading control. (C) Coomassie staining of purified proteins from HEK-293T/Flag-strepII-RFP (lane 1) and HEK-293T/Flag-strepII-SKAP (lane 2) cell lysates by TAP strategy. (D) SKAP interacting proteins were identified by mass spectrometry. The results are shown in the table.

To investigate the molecular mechanism of the pro-apoptotic role of SKAP, we employed a TAP/MS strategy to identify SKAP-interacting proteins in HEK-293T cells. We generated a cell line that stably expressed the Flag-strepII-SKAP recombinant protein and a control cell line that stably expressed the Flag-strepII-RFP recombinant protein (Figure 4A). Exogenous expression of Flag- and Strep-tagged SKAP or RFP was confirmed by immunoblot analysis (Figure 4B). Cellular extracts were prepared, subjected to successive affinity purification using Strep Tag II agarose and Flag agarose, and analyzed on an SDS-PAGE gel stained with Coomassie blue (Figure 4C). Mass spectrometric analysis showed that SKAP co-purified with four proteins, among which Prp19 was the most highly enriched (Figure 4D).

### SKAP Physically Associates with Prp19

**Figure 5:**
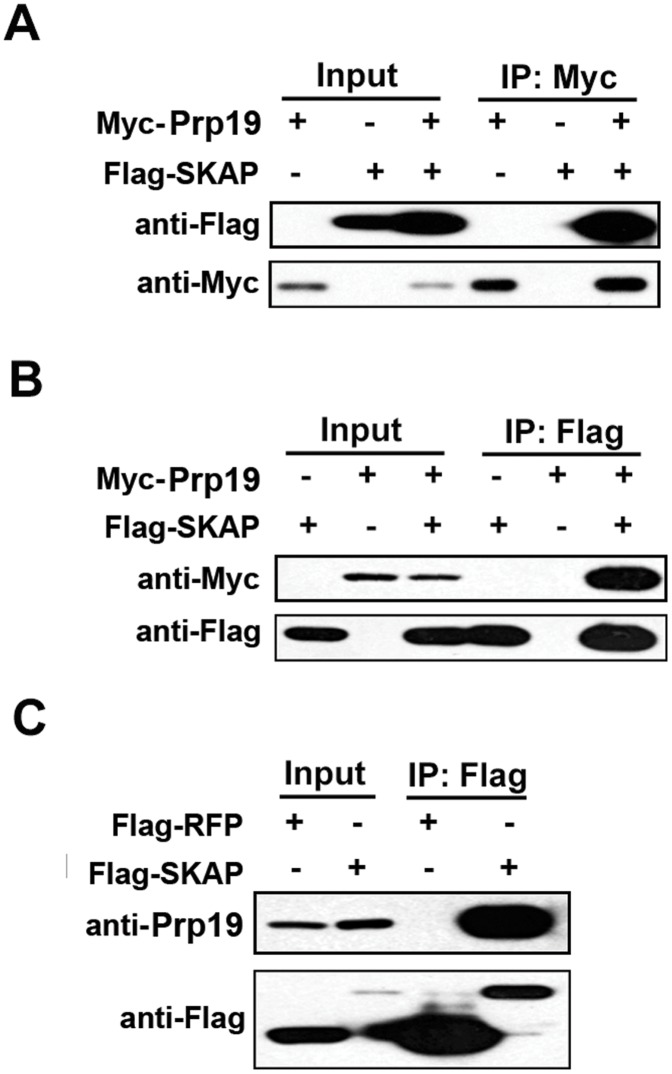
SKAP interacts with Prp19 *in vivo*. (A and B) Co-immunoprecipitation assays for SKAP and Prp19. HEK-293T cells were transfected with Flag-SKAP and Myc-Prp19. After immunoprecipitation with anti-Myc beads, Flag-SKAP was detected by immunoblotting using an anti-Flag antibody; after immunoprecipitation with anti-Flag beads, Myc-Prp19 was detected by immunoblotting using an anti-Myc antibody. (C) Immunoprecipitation assays for SKAP and Prp19. HEK-293T/Flag-strepII-RFP and HEK-293T/Flag-strepII-SKAP stable cell lysates were incubated with anti-Flag beads; endogenous Prp19 was detected by immunoblot using an anti-Prp19 antibody.

We next verified the interaction between SKAP and Prp19 by co-immunoprecipitation assays. Flag-SKAP and Myc-Prp19 were co-expressed in HEK-293T cells. Cellular extracts were immunoprecipitated with an anti-Myc antibody and analyzed by immunoblot with an anti-Flag antibody. As shown in Figure 5A, Flag-SKAP co-precipitated with Myc-Prp19. The interaction between Flag-SKAP and Myc-Prp19 was further confirmed in a reverse immunoprecipitation experiment (Figure 5B). We next sought evidence for the interaction of Flag-SKAP with endogenous Prp19. An immunoprecipitation assay showed that Flag-SKAP strongly and specifically associated with endogenous Prp19 in HEK-293T cells (Figure 5C). Taken together, these results illustrated that SKAP physically interacts with Prp19.

### Prp19 Suppresses UV-induced Cell Apoptosis

**Figure 6:**
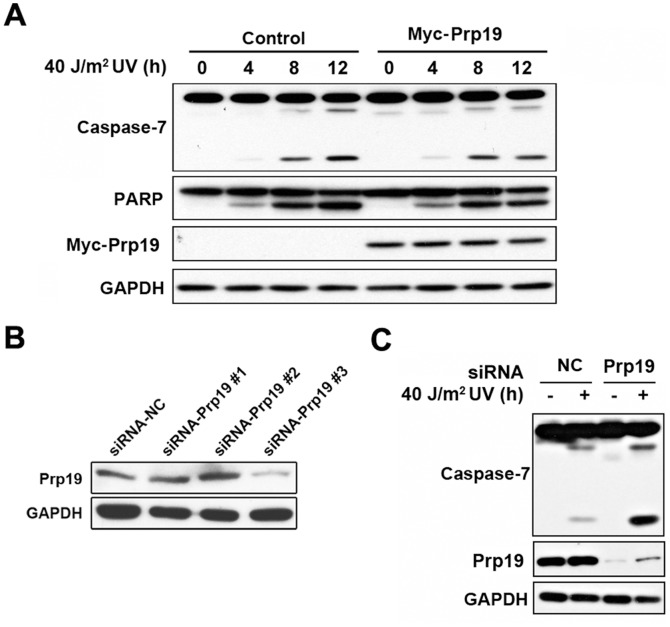
Prp19 suppresses UV-induced apoptosis in HeLa cells. (A) HeLa cells were transfected with pcDNA-Myc or pcDNA-Myc-Prp19 and treated with 40 J/m^2^ UV irradiation. The cell lysates were analyzed by immunoblotting with the antibodies indicated. GAPDH was used as a loading control. (B) The knockdown efficiency of siRNAs targeting Prp19 was verified by immunoblot analysis. (C) HeLa cells were transfected with siRNA-NC or siRNA-Prp19 #3 and treated with 40 J/m^2^ UV irradiation. The cell lysates were analyzed by immunoblotting with the antibodies indicated. GAPDH was used as a loading control.

Prp19 has been shown to inhibit cell apoptosis induced by hypoxic conditions [19] and the DNA damaging reagents methyl-methane sulfonate (MMS) and mitomycin C (MMC) [20], but the effect of Prp19 on UV-induced cell apoptosis had never been explored. To investigate this question, HeLa cells were transiently transfected with Prp19, exposed to UV irradiation, and apoptosis was detected by immunoblot analysis. As shown in Figure 6A, Prp19 overexpression reduced the levels of cleaved caspase-7 and cleaved PARP, indicating that Prp19 inhibits UV-induced apoptosis. To further confirm the anti-apoptotic role of Prp19, we performed a knockdown experiment using three siRNAs targeting Prp19. Immunoblot analysis showed that siRNA-Prp19 #3 could effectively inhibit Prp19 expression, and that construct was therefore used for subsequent experiments (Figure 6B). We next examined the effect of Prp19 knockdown on UV-induced cell apoptosis. Silencing of Prp19 significantly increased the levels of cleaved caspase-7 and cleaved PARP (Figure 6C). Taken together, the above findings suggest that Prp19 exerts an anti-apoptotic role in UV-induced apoptosis.

### SKAP Negatively Regulates the Protein Level of Prp19

**Figure 7:**
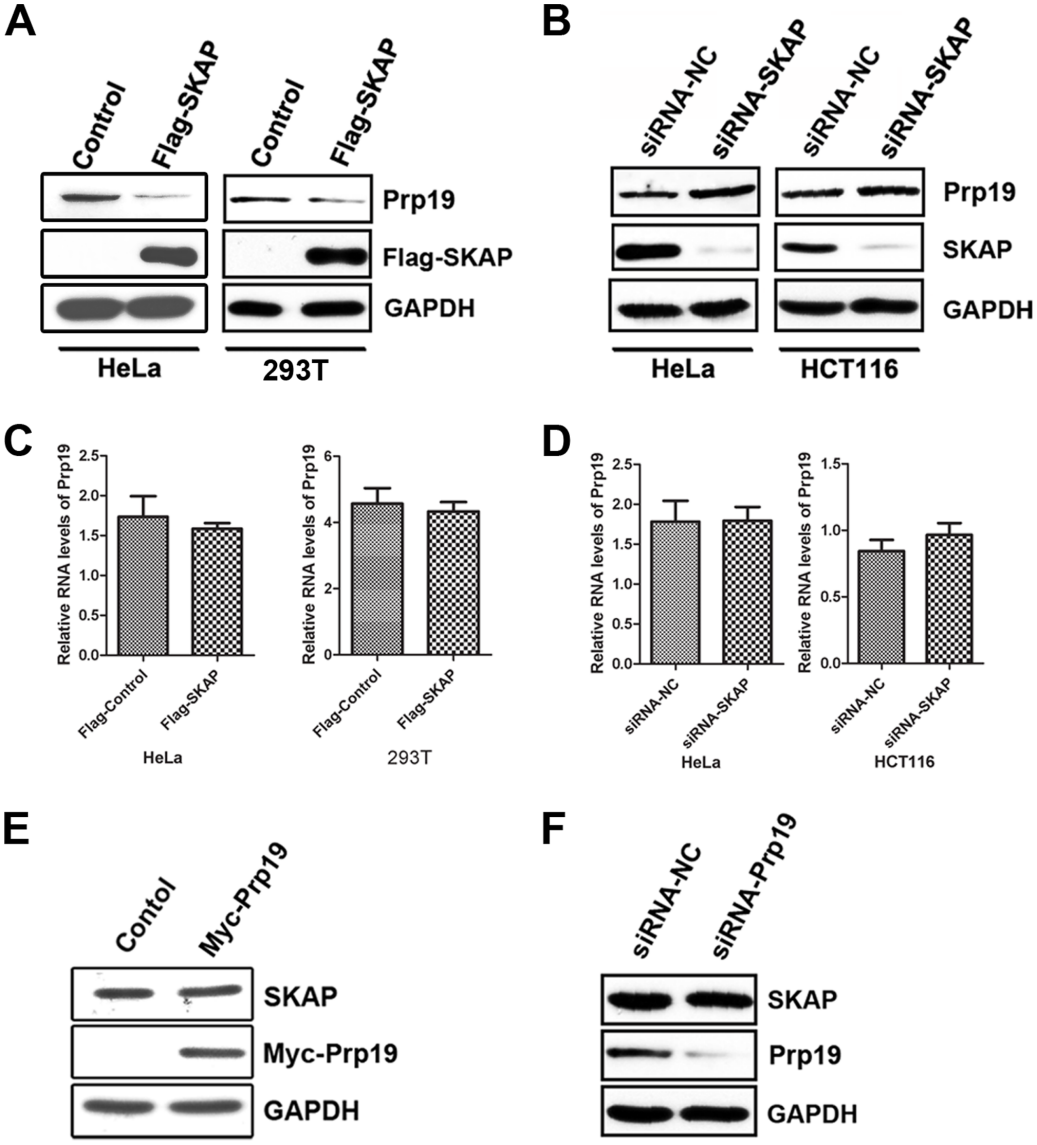
SKAP negatively regulates Prp19 protein levels. (A) HeLa and HEK-293T cells were transfected with pcDNA-Flag or pcDNA-Flag-SKAP and harvested after 36 h. The cell lysates were analyzed by immunoblotting with the indicated antibodies. GAPDH was used as a loading control. (B) HeLa and HCT116 cells were transfected with siRNA-NC or siRNA-SKAP #1 and harvested after 36 h. The cell lysates were analyzed by immunoblotting with the indicated antibodies. GAPDH was used as a loading control. (C) HeLa and HEK-293T cells were transfected with pcDNA-Flag or pcDNA-Flag-SKAP and harvested after 48 h, the relative RNA level of Prp19 were determined by qRT-PCR. Data were measured in triplicate, values and bars represent the mean and standard deviation, respectively. (D) HeLa and HCT116 cells were transfected with siRNA-NC or siRNA-SKAP #1 and harvested after 48 h, the relative RNA level of Prp19 were determined by qRT-PCR as described in (C). (E) HEK-293Tcells were transfected with pcDNA-Myc or pcDNA-Myc-Prp19 and harvested after 36 h. The cell lysates were analyzed by immunoblotting with the indicated antibodies. GAPDH was used as a loading control. (F) HeLa and HCT116 cells were transfected with siRNA-NC or siRNA-Prp19 #3 and harvested after 36 h. The cell lysates were analyzed by immunoblotting with the indicated antibodies. GAPDH was used as a loading control.

To further explore the molecular mechanism of SKAP in UV-induced apoptosis, we tested the relationship between SKAP and Prp19 beyond their interaction. HeLa and HEK-293T cells were transiently transfected with either Flag-SKAP or an empty vector, and cells were harvested after 36 h. Immunoblot analysis revealed that the protein level of Prp19 decreased upon ectopic expression of SKAP (Figure 7A), and the protein level of Prp19 increased when SKAP was silenced in HeLa and HCT116 cells (Figure 7B). We next examined whether SKAP regulated Prp19 gene transcription. As shown in Figure 7C and D, neither overexpression nor knockdown of SKAP changed the RNA level of Prp19. These results lead us to examine the feedback regulation of Prp19 on SKAP. We found that Prp19 overexpression in HEK-293T cells did not affect the protein level of SKAP (Figure 7E). Similarly, Prp19 knockdown in HeLa cells did not change the protein level of SKAP (Figure 7F). Collectively, these findings indicate that SKAP can negatively regulate the protein level of Prp19, whereas Prp19 does not alter SKAP expression.

### SKAP’s Pro-apoptotic Role Requires its Negative Regulation of Prp19

**Figure 8:**
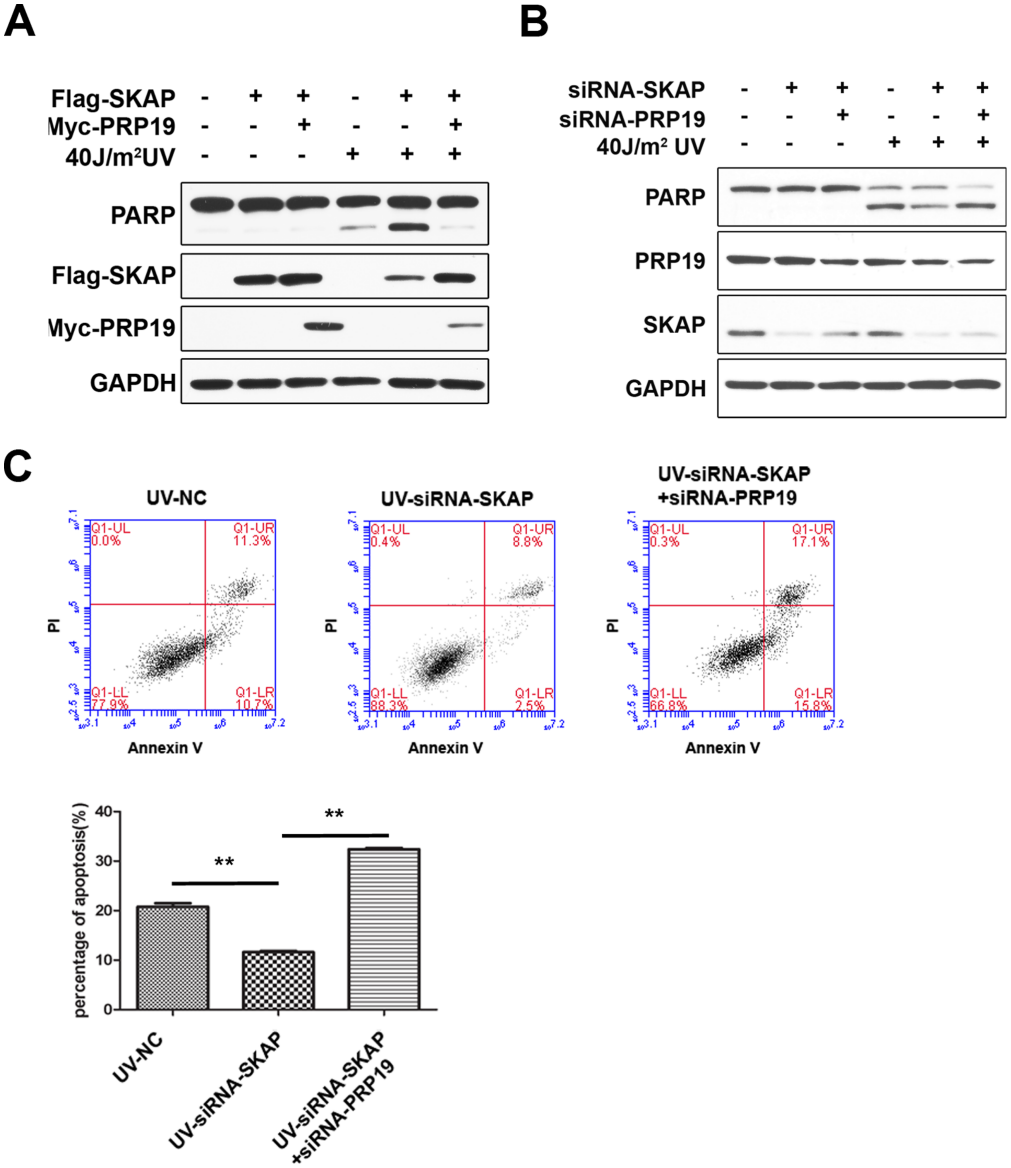
The pro-apoptotic role of SKAP is effected through its negatively regulation of Prp19. (A) HeLa/Flag-SKAP stable cells were transfected with pcDNA-Myc or pcDNA-Myc-prp19 and treated with 40 J/m^2^ UV irradiation. After 8 hours, the cell lysates were analyzed by immunoblot with the indicated antibodies. GAPDH was used as a loading control. (B) HeLa cells were transfected with siRNA-NC, siRNA-SKAP #1 or siRNA-SKAP #1 plus siRNA-Prp19 #3 and treated with 40 J/m^2^ UV irradiation. After 8 hours, the cell lysates were analyzed by immunoblot with the indicated antibodies. GAPDH was used as a loading control. (C) Annexin V/PI FACS analysis to analyze apoptosis in HeLa cells transfected with siRNA NC, siRNA-SKAP #1 or siRNA-SKAP #1 plus siRNA-Prp19 #3 and treated with 40 J/m^2^ UV irradiation. The percentage of apoptotic cells (% of total cells), including early apoptotic cells (Annexin V^+^, PI^−^) and late apoptotic cells (Annexin V^+^, PI^+^), is shown as a bar graph (*p<0.05). The data are representative of three different experiments, and the error bars represent the standard deviations of triplicate samples.

Based on the above results, we hypothesized that the regulation of cell apoptosis by SKAP may occur through suppression of Prp19. To test this hypothesis directly, we performed rescue experiments. HeLa cells stably expressing Flag-SKAP were transiently transfected with Myc-Prp19 or an empty vector for 30 h and then exposed to UV irradiation. Cells were collected after 12 h and analyzed by immunoblot analysis. The overexpression of SKAP promoted UV-induced cell apoptosis, and the pro-apoptotic effect of SKAP could be rescued by co-expression of Prp19 (Figure 8A). Next, we performed a rescue experiment using RNA interference. As illustrated in Figure 8B, SKAP knockdown decreased UV-induced cell apoptosis, and this effect could be rescued by simultaneous knockdown of Prp19. FACS analysis was performed to further confirm the results. As shown in Figure 8C, SKAP knockdown significantly decreased the percentage of apoptotic cells after UV exposure, and further knockdown of Prp19 restored the anti-apoptotic phenotype induced by SKAP knockdown (Figure 8C). Taken together, these data demonstrate that the pro-apoptotic role of SKAP is executed through negatively regulating Prp19.

## Discussion

Recent studies have shown that SKAP is a mitosis-associated protein that contributes to chromosome alignment, normal timing of sister chromatid segregation, and the maintenance of spindle pole architecture [12]–[16]. However, little is known about whether SKAP is involved in the regulation of cell apoptosis. In this study, we found that overexpression of SKAP promoted cell apoptosis after exposure to TNFα, TRAIL, Staurosporine and UV irradiation and that knockdown of SKAP had the opposite effect. These results demonstrate for the first time that SKAP has a pro-apoptotic effect on apoptotic stimuli triggering either the intrinsic or the extrinsic pathway.

Many cellular processes such as proliferation, differentiation and apoptosis are carried out by multi-protein complexes, and the identification of individual members of these complexes is pivotal for understanding their function. To investigate the mechanism by which SKAP influences cell apoptosis, we employed a tandem affinity purification (TAP) approach to search for protein partners that interact with SKAP. The TAP strategy is a two-step affinity purification scheme that generates protein complexes sufficiently clean to be analyzed by mass spectrometry. This approach allows the recovery of highly purified protein complexes present at very low concentration, and it is particularly well suited for the identification of interacting proteins. In our experiment, we incorporated a TAP tag (Flag-strepII tag) at the N-terminus of SKAP and stably expressed Flag-strepII-SKAP in HEK-293T cells by lentivirus-mediated infection. After a two-step purification and mass spectrometry analysis, we identified Prp19 as the best candidate partner. The SKAP-Prp19 interaction was further validated by immunoprecipitation assays. Taken together, our results provide the first evidence that SKAP forms a complex with Prp19 *in vivo*.

A key finding from our study is that SKAP can negatively regulate the level of Prp19 protein. Prp19 is highly evolutionarily conserved from yeast to humans [21]. It was first identified in a screen for mutants conferring sensitivity to X-rays, *psoralen*, and other interstrand cross-link (ICL) inducing agents [22]. Further characterization of this protein indicated that it was particularly sensitive to a broad range of DNA damaging agents, including IR, UV, monofunctional and bifunctional alkylating agents [20]. Increasing evidence has indicated that Prp19 is a multi-functional protein that participates in many physiological events, such as post-transcriptional regulation of eukaryotic genes [23]–[27], ubiquitin-proteasome degradation [28] and the DNA damage response [20], [29]. In our study, we revealed that Prp19 inhibits cell apoptosis after UV exposure, which supports current reports that Prp19 showed an anti-apoptotic effect under γ irradiation [29] and hypoxic conditions [19]. The contrasting effects of SKAP and Prp19 on apoptosis after UV irradiation made us further investigate the relationship between the expression levels of these two proteins. We found that SKAP could inversely regulate Prp19 expression at the protein level but not RNA level, suggesting that it’s a functional consequence of the SKAP-Prp19 interaction. However, our further study showed that Prp19 could not alter SKAP expression, which indicated that Prp19 was a downstream effector of SKAP.

Finally, we performed rescue experiments to prove that the pro-apoptotic role of SKAP is effected through Prp19. The results from both immunoblot analysis and FACS analysis revealed that Prp19 could significantly rescue the pro-apoptotic effect of SKAP after UV irradiation. Moreover, we noticed that the SKAP and Prp19 double knockdown group showed a higher degree of apoptosis than the control group in FACS analysis. We speculate that, in addition to being involved in SKAP-regulated apoptosis, Prp19 has a strong anti-apoptotic effect after UV irradiation, and more research should be conducted to explore Prp19’s intrinsic mechanism.

In conclusion, we discovered a novel role for SKAP in promoting cell apoptosis after UV irradiation. In addition, the anti-apoptotic protein Prp19 is associated with SKAP and antagonizes SKAP to regulate cell apoptosis. Our findings provide new insight to the understanding of SKAP’s function in the DNA damage response. Furthermore, based on a recent published result that the Prp19 complex directly functions in mitotic spindle assembly [30], we propose that the identification of the SKAP-Prp19 complex will help to further explore the role of SKAP in mitosis-related biological events.
